# ADHD Symptoms in Parents of Children With or Without ADHD: Prospective Association With Children’s Quality of Life and Emotional and Conduct Problems (EPINED Cohort)

**DOI:** 10.1007/s10802-026-01510-6

**Published:** 2026-09-23

**Authors:** Fátima El-Bouhali-Abdellaoui, Núria Voltas, Paula Morales-Hidalgo, Carmen Hernández-Martínez, Josefa Canals-Sans

**Affiliations:** 1 Nutrition and Mental Health Research Group (NUTRISAM), Universitat Rovira i Virgili (URV), Tarragona, Spain; 2 Department of Psychology, Faculty of Educational Sciences and Psychology, Universitat Rovira i Virgili, Carretera de Valls, s/n, 43007 Tarragona , Spain; 3 Research Center for Behavior Assessment (CRAMC), Department of Psychology, Universitat Rovira i Virgili (URV), Tarragona, Spain; 4 Institut d’Investigació Sanitària Pere Virgili (IISPV), Reus, Spain; 5 IU-RESCAT: University Research Institute on Sustainable, Climate Change and Energy Transition, Universitat Rovira I Virgili, Tarragona, Spain

**Keywords:** ADHD, Children emotional problems, Quality of Life, Parental ADHD symptoms

## Abstract

ADHD symptoms are frequently reported in parents of children with ADHD, however, their prevalence and association with children’s mental health and quality of life (QoL) remain poorly understood. This study examines parental ADHD symptoms and their associations with children’s mental health and QoL in the context of a prospective longitudinal study. The sample comprised 448 mothers and 375 fathers of 456 schoolchildren from the EPINED cohort (140 ADHD, 59 subthreshold ADHD symptoms, 257 comparison group). ADHD symptom rates were higher in mothers (18.2%) than in fathers (15.9%) and were significantly more frequent among mothers of children with ADHD (24.8%) compared with mothers in the comparison group (14.7%). Maternal ADHD risk (*OR* = 8.15) and maternal psychological difficulties (*OR* = 12.15), were both associated with the persistence of children’s emotional problems. Child ADHD diagnosis was associated with conduct problems (*OR* = 2.62) and their persistence (*OR* = 6.84). Additionally, maternal ADHD risk was consistently associated with poorer child QoL (*β*=-7.69). These findings suggest that maternal ADHD symptoms and psychological difficulties are key factors in children’s emotional outcomes, highlighting the need for family-focused assessment and intervention strategies.

**Supplementary Information:**

The online version contains supplementary material available at https://doi.org/10.1007/s10802-026-01510-6.

## Introduction

Attention-Deficit/Hyperactivity Disorder (ADHD) is one of the most common neurodevelopmental disorders characterized by persistent patterns of inattention and/or hyperactivity and impulsivity, which often manifest in childhood and can persist into adulthood (APA, 2013; Popit et al., 2024; Thöne et al., 2023). ADHD symptoms impact essential life domains, including social interactions, family functioning, and academic performance (Smit et al., 2021; Thöne et al., 2023) and the estimated global prevalence ranges from 1.6% to 5% (Popit et al., 2024). In Spain, the overall prevalence in the school-aged population is approximately 5%, with rates of 7.7% in school-age children and 3.0% in preschoolers (Canals Sans et al., 2021).

Although the exact cause remains unclear, ADHD is recognized as the result of interaction between genetic predisposition and early environmental influences (Al-Gailani & Al‐Kaleel, 2024; Faraone et al., 2021; Kaur et al., 2023). Twin and family studies have identified heritability estimates of approximately 70–80%, highlighting the significant role of genetic contributions in ADHD etiology (Demontis et al., 2023). Consistent with this strong genetic component, the adult prevalence of ADHD, which ranges between 1.4% and 4.6% in adults (Martin et al., 2025), is often reflected in familial studies. Research has consistently reported a high prevalence of ADHD symptoms among parents of children with ADHD, with estimates ranging from 41% to 55% (Schlarb et al. 2016; Takeda et al., 2010; Uchida et al., 2023), with approximately 40% in mothers and 50% in fathers (Schlarb et al., 2016; Uchida et al., 2023). Beyond shared genetic liability, a range of parenting and family environment factors have been associated with children’s ADHD outcomes, including parenting quality (e.g., family stress, chaos and co-parenting), adverse family experiences (e.g., maltreatment and parental separation), parental comorbid psychopathology, family socioeconomic disadvantage and broader sociocultural influences (Claussen et al., 2024; Flouri et al., 2017; Johnston & Chronis-Tuscano, 2017).

### Emotional and Psychopathological Comorbidities in ADHD

In addition to its core symptoms, ADHD is associated with an increased risk of comorbidities and psychopathological problems from childhood. Previous studies have reported that up to 38% of children diagnosed with ADHD have clinically significant symptoms of anxiety or depression (Mohammadi et al., 2021; Morales-Hidalgo et al., 2023). This emotional vulnerability appears to be further amplified in the presence of additional neurodevelopmental comorbidities, particularly given the high prevalence of autism-ADHD comorbidity, as estimated ranging between 38.5% and 40.2% (Rong et al., 2021). Specifically, the proportion of children with anxiety or depressive symptoms rises to 50% in those with subthreshold symptoms of ADHD (without clinical diagnosis) and reaches 57% among children with a comorbid diagnosis of ADHD + Autism (Morales-Hidalgo et al., 2023). This high comorbidity burden extends into adulthood, where ADHD frequently co-occurs with anxiety and mood disorders (Fu et al., 2025; Gair et al., 2021)These associations may emerge as a result of the functional impact of ADHD on different areas of life (Maxwell et al., 2025; Turjeman-Levi et al., 2024). However, they may also be linked to ADHD-related characteristic, such as emotion dysregulation, which have been associated with a greater risk of psychopathology throughout the lifespan (Beheshti et al., 2020).

### Parental ADHD and Child Outcomes

While ADHD in children is known to have a broad impact on behavioral and emotional functioning, the prevalence of parental ADHD symptoms and their influence on children’s lives over time have not been sufficiently investigated in longitudinal studies. Cross-sectional studies in clinical samples have shown that children with ADHD whose parents also have ADHD tend to exhibit greater symptom severity (Takeda et al., 2010), worse neurocognitive functioning (Agha et al., 2020), and are exposed to more negative parenting practices (Smit et al., 2021). Relatively few studies have examined whether parental ADHD symptoms in population-based cohorts (including children with and without ADHD) predict children’s emotional problems and quality of life beyond child ADHD diagnostic status, and co-occurring parental psychological distress. Moreover, many previous studies have not accounted for common neurodevelopmental comorbidities (e.g. autism) which limit the generalizability to clinically meaningful diagnostic profiles in children. Furthermore, comparison groups of children whose parents do not report ADHD are uncommon, and maternal and paternal ADHD symptoms are rarely examined separately, despite evidence suggesting potentially differential effects on child outcomes by parent sex (Takeda et al., 2010).

### The Present Study

Using a prospective longitudinal design within a school-based epidemiological cohort, the primary aim of this study is to examine whether maternal and paternal ADHD symptoms, considered separately, constitute independent family-level risk factors for children’s emotional and conduct problems and quality of life. These associations were analyzed while accounting for child ADHD, parental psychological distress, and relevant sociodemographic and cognitive covariates. We further examined whether parental ADHD symptoms are associated with the persistence of children’s emotional and conduct problems at two time points during development, and whether these associations differ according to parental sex. As a secondary aim, we estimated the frequency of ADHD symptoms in mothers and fathers and compare it across child diagnostic groups (ADHD diagnosis, subthreshold ADHD symptoms and ADHD comorbid with autism), and children without ADHD or autism.

We hypothesized that higher parental ADHD symptoms would be prospectively associated with greater emotional and conduct problems and lower perceived quality of life in children, independently of parental psychological distress. We also expected that these associations would vary by parental sex. Finally, we hypothesized that both mothers and fathers of children with ADHD or subthreshold ADHD symptoms would report higher ADHD symptom levels than those in the comparison group.

## Methods

### Study Design

This study is part of a prospective longitudinal research project that aimed to explore the prevalence of ADHD and autism in a representative sample of school children from the province of Tarragona (Catalonia, Spain) at two different developmental stages (preschoolers of 4–5 years and primary school children of 10–11 years). Data for the first assessment point were obtained from the Neurodevelopmental Disorders Epidemiological Research Project (EPINED) and were collected between 2014 and 2019. The study protocol was validated by the Ethics Committee at the Sant Joan University Hospital (*13-10-31/10proj5*). EPINED had an initial screening phase where parents (*n* = 3,727) and teachers (*n* = 6,894) completed questionnaires for the detection of ADHD and autism. A total of 3,727 children had responses from both parents and teachers (1,802 preschoolers and 1,925 primary schoolchildren). Of these, 781 participants (484 identified as at risk for ADHD and/or autism, and 297 without symptoms of either condition) advanced to the second phase, where they were individually assessed by trained clinicians to establish a diagnosis of ADHD and/or autism according to DSM-5 criteria. Further details on this project can be found in (Canals Sans et al., 2021) and (Morales Hidalgo et al., 2021).

The second assessment point corresponded to EPINED 2, the follow-up project involving participants who had completed the second phase of EPINED, with data collected between 2020 and 2023. The aim of EPINED 2 was to examine the clinical course of children previously diagnosed with ADHD and/or autism, those presenting symptoms without meeting diagnostic criteria, and children without a diagnosis of either condition. For this purpose, in the first phase of the EPINED 2, 493 parents and 501 teachers completed a battery of psychological tests to assess symptoms of ADHD, autism, and emotional and conduct disorders. In the second phase of EPINED 2, individual assessments were conducted with participants who had a prior diagnosis of ADHD or autism, who were identified as having past or current risk for these conditions, or who showed emotional symptoms in the first phase of EPINED 2.

Written informed consent was obtained from parents, and assent was obtained from children, at the initial screening phase of both EPINED and EPINED 2, prior to any data collection.

### Children’s Psychological Assessment and Diagnostic Procedure

During EPINED, sociodemographic and clinical data were gathered using a custom-designed questionnaire., and socioeconomic status was based on the Hollingshead index (Hollingshead, 2011). ADHD risk was screened using parent- and teacher-reported Conners Index versions (Conners & Goldstein, 2009; Conners, 2008), whereas autism risk was assessed through the parent-reported Childhood Autism Spectrum Test (CAST), completed by parents (Morales-Hidalgo et al., 2017b; Scott et al., 2002) and the teacher-reported EDUTEA questionnaire (Morales-Hidalgo et al., 2017a, ). In the second phase, ADHD and autism diagnoses were evaluated the Schedule for Affective Disorders and Schizophrenia for School-Aged Children –Present and Lifetime version (K-SADS-PL; Kaufman et al., 1997) and the Autism Diagnostic Interview-Revised (ADI-R; Rutter et al., 2003) interviews, respectively, complemented by direct child assessment with the Autism Diagnostic Observation Schedule, Second Edition (ADOS-2; Lord et al., 2012). Cognitive functioning was assessed as age-appropriate Wechsler scales (WPPSI-IV; Wechsler, 2014 and WISC-IV; Wechsler et al., 2005). In addition, parents completed the Child Behavior Checklist (CBCL 1½–5 or CBCL/6–18; Achenbach & Rescorla, 2000, 2001) to screen for comorbid psychological symptoms. The CBCL provides scores on syndrome, broad-band, and DSM-5–oriented scales. For this study, we used the Internalizing and Externalizing broad-band scales. *T*-scores were derived from Spanish norms, with scores between 60 and 63 considered borderline and those ≥ 64 within the clinical range. Clinical and educational information provided by parents, children, and, when applicable, by the school psychology team, together with neuropsychological profile data obtained directly from the children, was also considered for the diagnoses.

During EPINED 2, parents completed the Conners ADHD Rating Scale 3 Parent Short Form (Conners & Goldstein, 2009; Conners, 2008) and the original Spanish version of the Social Responsiveness Scale 2nd Edition (SRS-2; Constantino & Gruber, 2012), which measures autistic traits across social communication/interaction and restricted/repetitive behaviors to detect new cases of risk of ADHD or autism. They also completed the validated Spanish version of the Strengths and Difficulties Questionnaire (SDQ; Goodman, 1997; Ortuño-Sierra et al., 2015). This 25-item instrument measures emotional and behaviour adjustment through five subscales (Emotional difficulties, Conduct difficulties, Hyperactivity, Peer difficulties, and Prosocial). For this study, analyses focused on the Emotional and Conduct difficulties subscales. According to parent-rated scoring cut-offs, scores on Emotional difficulties indicate borderline (4) or clinical (≥ 5) levels, while scores of 3 and above on the Conduct difficulties indicate borderline (3) or clinical (≥ 4) levels.

In the second phase of the EPINED 2, diagnostic procedures followed the same DSM-5 criteria and standardized tools for ADHD (K-SADS-PL) and for autism (ADOS-2 and the autism module of the K-SADS-PL).

Parents also completed the Spanish version of the KIDSCREEN-27 (Pardo-Guijarro et al., 2013; Ravens-Sieberer et al., 2007) to evaluate QoL of their children, a 27 item instrument which covers five dimensions of health-related well-being (Physical Well-Being, Psychological Well-Being, Autonomy and Parent Relations, Peers and Social Support, and School Environment). Items are rated on a 5-point Likert scale (from 1 =” not at all” to 5 =” very much”) and demonstrated good internal consistency (Cronbach’s *α* = 0.82; Pardo-Guijarro et al., 2013). For the present analysis, a global QoL score was computed by summing all the items (after reverse scoring items 1, 9, 10 and 11), since the instrument does not provide an official total score. Higher total scores reflected better perceived QoL (Budler et al., 2022).

### Parental Self-reported Psychological Assessment

In both EPINED and EPINED 2, parents also completed the Spanish versions of the General Health Questionnaire Short Form (GHQ-12; Goldberg & Hillier, 1979; Del Pilar Sánchez-López & Dresch, 2008) and the Adult ADHD Self-Report Scale (ASRS; Kessler et al., 2007; Ramos Quiroga et al., 2009). The GHQ-12 consists of 12 items rated on a 4-point Likert scale (0–3), with a total score ≥ 12 used to identify probable psychological difficulties (Cronbach’s *α* = 0.78; Rathore et al., 2022). The ASRS includes 18 items aligned with DSM-5 criteria, rated on a 5-point Likert scale, and divided into two domains: inattention (9 items) and hyperactivity/impulsivity (9 items). A cutoff score of ≥ 4 on the first six screening items suggests possible ADHD (Cronbach’s *α* = 0.68), while for the full 18-item scale, shows high internal consistency (Cronbach’s *α* = 0.92; Pedrero Pérez & Puerta García, 2007). A score ≥ 6 in either domain indicates a positive screen for that symptom dimension (Xia et al., 2015).

For the present study, ASRS data from EPINED were used. Missing ASRS data (*n* = 81 for mothers and *n* = 63 for fathers) were handled through longitudinal substitution of observed values within participants, using ASRS scores from EPINED 2 when available. This approach was supported by the high correlations observed among participants who completed both waves (*r* = .642 and 0.696 respectively, *p*<.001), indicating strong temporal stability of ASRS scores.

### Case Definitions

In both phases of the project (EPINED and EPINED 2), diagnostic assessments of ADHD and autism were conducted individually with children and their families at the schools, following DSM-5 criteria and using standardized clinical interviews and observational measures. Children were classified into the ADHD group when DSM-5 diagnostic criteria for any of the three ADHD presentations were fulfilled. A separate subthreshold ADHD symptom group included children who showed ADHD-related symptoms on the K-SADS-PL but did not meet the full diagnostic threshold. This group was defined by meeting 4 or 5 (depending on whether the participants were children or older adolescents) inattentive or hyperactive-impulsive diagnostic criteria on the K-SADS-PL, showing positive screening results in both parent and teacher Conners reports (*T* ≥ 65), and presenting considerable impairment in daily life. The comparison group consisted of children without ADHD or autism diagnoses or risk for either condition, although some presented emotional or conduct problems.

Persistence of emotional and conduct problems was defined as the presence of clinical-range scores on the corresponding CBCL scales (Internalizing and Externalizing) in EPINED and on the emotional and conduct difficulties subscales of the SDQ in EPINED 2. Children who did not meet the clinical cut-off at both time points were classified as non-persistent.

### Participants

The present study included 456 children (268 boys and 188 girls), as well as 448 mothers and 375 fathers. Children were classified into four diagnostic groups: (1) 127 with ADHD, (2) 59 with subthreshold ADHD symptoms, (3) 13 with comorbid ADHD and autism (hereafter, ADHD + Autism), and (4) 257 in a comparison group without ADHD or autism. The most common ADHD presentation was the combined type (47.2%), followed by the inattentive (37.2%) and hyperactive/impulsive (15.6%) presentations. Children in the ADHD groups (ADHD and subthreshold ADHD symptoms) were predominantly male (116 boys and 70 girls) and had a mean age of 13.6 years (*SD* = 2.9).

### Statistical Analyses

Statistical analyses were conducted using IBM SPSS Statistics (v29.0.1) and EPIDAT (v4.1.). Descriptive statistics were calculated to characterize the study sample, with continuous variables indicated as means and standard deviations (*SD*) and categorical ones as frequencies (*n*) and percentages (%). To examine differences in sociodemographic, clinical and psychological characteristics across ADHD condition groups (ADHD, subthreshold ADHD symptoms and ADHD with comorbid autism) and the comparison group, inferential analyses were selected according to the type of variable. Group differences in categorical variables, including ASRS and GHQ-12 risk status as well as clinical classifications based on CBCL (Internalizing and Externalizing domains) and SDQ (Emotional and Conduct difficulties), were assessed using chi-square tests, followed by Z-tests for independent proportions when the results were statistically significant. Differences in continuous variables, including age, Intelligence Quotient (IQ), and total scores on the KIDSCREEN-27, ASRS and GHQ-12, were analyzed using one-way analysis of variance (ANOVA) with Bonferroni-adjusted post-hoc comparisons. Associations between parental ADHD symptoms and parental mental health were analyzed with chi-square and Student’s t test (see Supplementary Table 1). To examine the bivariate associations among the continuous variables included in the analyses, Pearson correlations were computed (see Supplementary Table 2).

Multivariable regression analyses using Enter method were performed to assess the association between maternal and paternal ADHD symptoms and children’s emotional and conduct problems at follow-up, as well as the persistence of these problems over time and their QoL. Logistic regression models were applied when the outcomes variables were defined categorically. Specifically, validated cut-off points were applied to the emotional and conduct measures to establish clinically significant risk profiles (i.e., the presence of emotional or conduct problems at follow-up, based on SDQ clinical cut-off scores, and the persistence of these problems across the study waves, based on both CBCL and SDQ clinical cut-off scores). On the other hand, multiple linear regression models were used when the outcome was the total score on the children’s parent-reported QoL (KIDSCREEN-27). The regressions starting with an unadjusted model (Model 1) including a dichotomous parental ADHD risk based on the ASRS cut-off (0 = without ADHD risk, 1 = with ADHD risk); and sequentially adjusting by sociodemographic, and cognitive covariates, and two dummy-coded variables to examine the differential effects of ADHD diagnosis and ADHD symptoms (Model 2). One of the dummy variables represented a clinical ADHD diagnosis (1 = diagnosis, including children with comorbid autism; 0 = no diagnosis, including children with subthreshold ADHD symptoms), and the other one dummy variable represented subthreshold ADHD symptoms without a formal diagnosis (1 = subthreshold symptoms without diagnosis; 0 = no symptoms, including diagnosed cases). Subsequently, Model 3 included all variables from Model 2 and parental psychological difficulties risk based on the GHQ-12 cut-off (0 = without ADHD risk, 1 = with ADHD risk). Finally, when emotional problems were the outcome, Model 4 was conducted including all variables from Model 3 adding the interaction term between maternal ADHD risk and maternal psychological difficulties risk to examine their combined contribution after a significant main effect of maternal psychological difficulties had been observed.

## Results

### Parents’ and Children’s Sociodemographic and Psychological Characteristics

Parents’ and children’s sociodemographic characteristics by ADHD condition (ADHD, ADHD symptoms, ADHD + Autism) are presented in Table 1. For the whole sample, the mean age of the mothers was 39 (*SD* = 5.9) and for the fathers it was 41.7 (*SD* = 6.8) (results not shown in the table). Children with ADHD and subthreshold ADHD symptoms had a significantly higher proportion of separated parents than those in the comparison group. Similarly, families of children with ADHD and subthreshold ADHD symptoms showed a higher prevalence of low socioeconomic status.

**Table 1:** Sociodemographic and psychological characteristics of the sample (*N* = 456)

		Comparison^a^	ADHD Conditions			*p*	Group Difference
			ADHD^b^	Subthreshold ADHD Symptoms^c^	ADHD + Autism^d^		
*(n)*		*257*	*127*	*59*	*13*		
Parents							
*Sociodemographic characteristics*							
Age, years, *M*(*SD*)							
Mothers		39.2(5.7)	39.1(5.8)	38.0(5.6)	41.3(3.8)	0.240	
Fathers		41.9(5.9)	41.9(6.3)	41.5(6.8)	41.9(4.5)	0.989	
Parents’ relationship, %(*n*)							
Separated		19.1(49)	33.9(43)	37.3(22)	15.4(2)	**0.034**	ab***, ac**
Family socioeconomic status, %(*n*)							
Low		11.7(27)	26.8(34)	23.7(14)	15.4(2)	**0.004**	ab***, ac**
Medium		68.1(175)	61.4(78)	55.9(33)	53.8(7)		
High		20.2(52)	11.8(15)	20.3(12)	30.8(4)		ab*, cd**
*Psychological Assessment*							
ASRS scale							
Mothers		*n* = 251	*n* = 125	*n* = 59	*n* = 13		
Risk (≥ 4), % (*n*)		14.7(37)	24.8(31)	15.3(9)	46.2(6)	**0.005**	ab*, ad**
Fathers		*n* = 216	*n* = 95	*n* = 44	*n* = 10		
Risk (≥ 4), % (*n*)		15.7(34)	15.8(15)	15.9(7)	20.0(2)	0.988	
GHQ-12 Scale							
Mothers		*n* = 253	*n* = 125	*n* = 58	*n* = 12		
Total Scale (≥ 12), % (*n*)		20.6(52)	37.6(47)	36.2(21)	41.7(5)	**0.001**	ab***, ac*
Fathers		*n* = 220	*n* = 96	*n* = 49	*n* = 10		
Total Scale (≥ 12), % (*n*)		16.8(37)	27.1(26)	20.4(10)	50.0(5)	**0.022**	ab*, ad**, cd*
Children							
*Sociodemographic characteristics*							
Age, *M*(*SD*)							
Years		13.2(3.0)	14.2(2.8)	14.1(2.9)	13.7(2.9)	**0.034**	ab*
Sex, %(*n*)							
Male		54.9(141)	66.1(84)	54.2(32)	84.6(11)	**0.036**	ac*, ad*, cd*
Female		45.1(116)	33.9(43)	45.8(27)	15.4(2)		
*Psychological Assessment*							
ADHD subtype, %(*n*)							
Inattentive		-	34.6(44)	50.8(30)	0.0(0)	**< 0.001**	bc*
Hyperactive/Impulsive		-	11.8(15)	20.3(12)	30.8(4)		
Combined		-	53.5(68)	28.8(17)	69.2(9)		bc**, bd**
*Cognitive Assessment*							
Total Wechsler IQ, *M*(*SD*)		102.,2(14.9)	94.8(13.0)	99.7(13.8)	91.0(16.9)	**< 0.001**	ab***, ad*
*Psychological Problems*							
EPINED	**CBCL scale**	*n* = 253	*n* = 126	*n* = 58	*n* = 13		
	**Internalizing problems**						
	Risk (*T-*score ≥ 64), % (*n*)	19.0(48)	46.0(58)	32.8(19)	61.5(8)	**< 0.001**	ab***, ac*, ad***
	**Externalizing problems**						
	Risk (*T-score* ≥ 64), % (*n*)	11.5(29)	48.4(61)	22.4(13)	61.5(8)	**< 0.001**	ab***, ac*, ad***, bc***, cd**
EPINED 2	**SDQ scale**	*n* = 200	*n* = 93	*n* = 43	*n* = 13		
	**Emotional difficulties**						
	Risk (*T-score* ≥ 5), % (*n*)	21.0(42)	26.9(25)	14.0(6)	46.2(6)	0.065	ad*, cd*
	**Conduct difficulties**						
	Risk (*T-score* ≥ 4), % (*n*)	16.5(33)	34.4(32)	23.3(10)	46.2(6)	**0.001**	ab***, ad**
Persistent problems, %(*n*)		*n* = 197	*n* = 92	*n* = 42	*n* = 13		
Emotional problems		8.6(17)	17.4(16)	7.1(3)	15.4(2)	0.120	ab*
Conduct problems		6.1(12)	26.1(24)	7.1(3)	38.5(5)	**< 0.001**	ab***, ad***, bc*, cd**
*Quality of life*							
KIDSCREEN-27, *M*(*SD*)		*n* = 82	*n* = 64	*n* = 23	*n* = 10		
Total scale		107.4(14.5)	106.4(15.3)	109.8(9.3)	98.9(10.3)	0.223	

*ADHD* Attention-Deficit/Hyperactivity Disorder, *M* Mean, *SD *Standard Deviation, *ASRS *Adult ADHD Self-Report Scale, *GHQ-12 *General Health Questionnaire Short Form,* IQ* Intelligence Quotient, *CBCL *Child Behavior Checklist, *SDQ* Strengths and Difficulties Questionnaire. SDQ emotional difficulties: positive risk on the emotional difficulties’ subscale; SDQ conduct difficulties: positive risk on the conduct difficulties subscale; Persistent problems: both CBCL and SDQ clinical risk. Data sources: EPINED: Baseline assessment, EPINED 2 = follow-up; CBCL, GHQ-12, ASRS, and IQ data correspond to EPINED assessment, while SDQ and KIDSCREEN data correspond to EPINED 2 assessment. Significant differences: *for *p <* .05, ** for *p*⩽0.01 and *** for *p*⩽0.001.

Regarding parents’ psychological assessment, 18.2% of mothers (*n* = 83) and 15.9% of fathers (*n* = 58) met criteria for ADHD risk (*p*≤.01) based on the ASRS cut-off. Overall, 28.2% (*n* = 105) of the children had at least one parent scoring above the cut-off for ADHD symptoms, while 4.8% (*n* = 18) had both parents scoring above this threshold. Parents of children in the ADHD groups reported higher levels of ADHD symptoms and psychological difficulties than those in the comparison group (Table 1). Specifically, there is a significantly higher proportion of mothers with ADHD risk in the ADHD + Autism (46.2%) and ADHD (24.8%) groups than in the comparison group (14.7%). Regarding psychological difficulties, parents of children in the ADHD groups reported higher rates. Parents identified as at risk for ADHD reported higher levels of psychological difficulties than parents without ADHD risk (see Supplementary Table 1).

### Emotional Problems in Children by the Presence of Parental ADHD risk

As presented in Table 2, logistic regression analyses showed that maternal ADHD risk was consistently associated with children’s emotional problems at follow-up (EPINED 2) and with their persistence across EPINED and EPINED 2. However, once maternal psychological difficulties were included in the models, they emerged as the only factor associated with children’s emotional problems at follow-up and with its persistence. When the interaction between maternal ADHD risk and maternal psychological difficulties was added in the last Model (Model 4), maternal psychological difficulties remained associated with children’s emotional problems at follow-up (*OR* = 4.304; 95% *CI* [1.935, 9.570]), whereas both maternal ADHD risk and maternal psychological difficulties were independently and strongly associated with children’s emotional problems persistence over time (*OR* = 8.152; 95% *CI* [1.738, 38.242]; *OR* = 12.154; 95% *CI* [3.525, 41.903], respectively). Fathers’ outcomes were not significantly associated with children’s emotional problems, either at follow-up or with their persistence over time, across all models.

**Table 2:** Association between children’s emotional problems and parental ADHD risk across two assessment points

	Emotional problems	
	Presence at EPINED 2	Persistence across EPINED and EPINED 2
	*OR* (95% *CI*)	*OR* (95% *CI*)
Unadjusted Models		
Model 1		
Mother’s ADHD risk	2.856 (1.390, 5.867)**	4.307 (1.772, 10.472)***
Father’s ADHD risk	1.469 (0.700, 3.082)	1.734(0.657, 4.574)
	R^2^ Nagelkerke = 0.053	R^2^ Nagelkerke = 0.088
	*p*=.010	*p*=.003
Adjusted Models		
Model 2		
Mother’s ADHD risk	2.465 (1.166, 5.212)*	3.697 (1.462, 9.348)**
Father’s ADHD risk	1.610 (0.746, 3.474)	1.804 (0.651, 4.997)
Dummy ADHD	1.646 (0.828, 3.272)	3.197 (1.195, 8.551)*
Dummy subthreshold ADHD symptoms	0.387 (0.107, 1.402)	1.616 (0.394, 6.628)
	R^2^ Nagelkerke = 0.116	R^2^ Nagelkerke = 0.152
	*p*=.007	*p*=.011
Model 3		
Mother’s ADHD risk	1.881 (0.842, 4.201)	2.395 (0.875, 6.558)
Father’s ADHD risk	1.692 (0.756, 3.790)	1.767 (0.600, 5.199)
Dummy ADHD	1.426 (0.694, 2.929)	2.717 (0.942, 7.831)
Dummy subthreshold ADHD symptoms	0.280 (0.073, 1.065)	1.126 (0.249, 5.095)
Mother’s psychological difficulties	4.085 (1.979, 8.433)***	6.508 (2.435, 17.396)***
Father’s psychological difficulties	0.958 (0.423, 2.170)	4.511 (0.541, 4.222)
	R^2^ Nagelkerke = 0.199	R^2^ Nagelkerke = 0.285
	*p*<.001	*p*<.001
Model 4		
Mother’s ADHD risk	2.102 (0.720, 6.137)	8.152 (1.738, 38.242)**
Father’s ADHD risk	1.683 (0.751, 3.774)	1.659 (0.558, 4.933)
Dummy ADHD	1.426 (0.695, 2.927)	2.590 (0.900, 7.451)
Dummy subthreshold ADHD symptoms	0.281 (0.074, 1.069)	1.196 (0.266, 5.382)
Mother’s psychological difficulties	4.304 (1.935, 9.570)***	12.154 (3.525, 41.903)***
Father’s psychological difficulties	0.966 (0.427, 2.186)	1.566 (0.565, 4.338)
Mother’s interaction between ADHD risk and psychological difficulties	0.785 (0.164, 3.754)	0.141 (0.019, 1.023)
	R^2^ Nagelkerke = 0.199	R^2^ Nagelkerke = 0.311
	*p*<.001	*p*<.001

Logistic regression. Models 2–4: adjusted for sex, age, family socioeconomic status, and total IQ. IQ: Intelligence Quotient; ADHD: Attention-Deficit/Hyperactivity Disorder. Cut-off scales (0 = below cut-off; 1 = at or above cut-off): parental psychological problems (GHQ-12 ≥ 12) and ADHD risk (ASRS ≥ 4). Dummy ADHD: ADHD (1) and subthreshold ADHD symptoms, comparison group (0); Dummy subthreshold ADHD symptoms: subthreshold ADHD symptoms (1) and ADHD, comparison group (0). Data sources: EPINED: Baseline assessment, EPINED 2 = follow-up. Significant differences: *for *p* < .05, ** for *p*⩽0.01 and *** for p⩽0.001

### Conduct Problems of Children by Parental ADHD Risk

Table 3 presents the results of the logistic regression models predicting the risk of child conduct problems at both cross-sectional and longitudinal assessments. Parental ADHD risk and emotional problems were not significantly associated with children’s conduct problems at EPINED 2. Although maternal ADHD risk predicted problem persistence in the unadjusted model, this effect was no longer significant after adjusting for other variables. Father’s ADHD risk was not significantly associated with children’s conduct problems at follow-up or with their persistence, in any model. Children’s ADHD was the only variable significantly associated with the conduct problems at the follow-up (*OR* = 2.620; 95% *CI* [1.264–5.429]) and with their persistence (*OR* = 6.841; 95% *CI* [2.560-18.278]) across both times.

**Table 3:** Association between children’s conduct problems and parental ADHD risk across two assessment points

	Conduct problems	
	Presence at EPINED 2	Persistence across EPINED and EPINED 2
	*OR* (95% CI)	*OR* (95% CI)
Unadjusted Models		
Model 1		
Mother ADHD risk	1.399 (0.647, 3.023)	2.522(1.058, 6.014)*
Father ADHD risk	1.560 (0.754, 3.229)	1.666(0.683, 4.064)
	R^2^ Nagelkerke = 0.013	R^2^ Nagelkerke = 0.041
	*p*=.319	*p*=.059
Adjusted Models		
Model 2		
Mother’s ADHD risk	1.050 (0.456, 2.419)	1.584(0.587, 4.276)
Father’s ADHD risk	1.514 (0.701, 3.271)	1.764(0.643, 4.834)
Dummy ADHD	2.958 (1.452, 6.027)**	7.010(2.693, 18.245)***
Dummy subthreshold ADHD symptoms	1.341 (0.480, 3.743)	1.317(0.256, 6.760)
	R^2^ Nagelkerke = 0.136	R^2^ Nagelkerke = 0.270
	*p*=.002	*p*<.001
Model 3		
Mother’s ADHD risk	0.878 (0.370, 2.086)	1.528 (0.556, 4.200)
Father’s ADHD risk	1.518 (0.699, 3.297)	1.741 (0.633, 4.786)
Dummy ADHD	2.620 (1.264, 5.429)**	6.841 (2.560, 18.278)***
Dummy subthreshold ADHD symptoms	1.139 (0.400, 3.244)	1.312 (0.251, 6.867)
Mother’s psychological difficulties	1.971 (0.938, 4.141)	0.916 (0.330, 2.544)
Father’s psychological difficulties	1.171 (0.521, 2.631)	1.384 (0.485, 3.951)
	R^2^ Nagelkerke = 0.157	R^2^ Nagelkerke = 0.272
	*p*=.001	*p*<.001

Logistic regression. Models 2–3: adjusted for sex, age, family socioeconomic status, and total IQ. IQ: Intelligence Quotient; ADHD: Attention-Deficit/Hyperactivity Disorder. Cut-off scales (0 = below cut-off; 1 = at or above cut-off): parental psychological problems (GHQ-12 ≥ 12) and ADHD risk (ASRS ≥ 4). Dummy ADHD: ADHD (1) and subthreshold ADHD symptoms, comparison group (0); Dummy subthreshold ADHD symptoms: subthreshold ADHD symptoms (1) and ADHD, comparison group (0). Data sources: EPINED: Baseline assessment, EPINED 2 = follow-up. Significant differences: *for *p* < .05, ** for p⩽0.01 and *** for p⩽0.001. All models are adjusted for sex, age, family socioeconomic status, and total IQ

### Quality of Life and Emotional State of Children by the Presence of Parental ADHD Risk

Descriptively, no statistically significant differences in parent-reported children’s QoL were observed between groups, although the ADHD + Autism group showed the lowest mean score (see Table 1).

As shown in Table 4, maternal ADHD risk was consistently and significantly associated with children’s QoL across all linear regression models (*p* < .05). In contrast, paternal ADHD risk was not significantly associated with children’s QoL in any of the regression models.

**Table 4:** Association between children’s quality of life and parental ADHD risk

	Children’s Quality of Life
	Total Score
	*β* (95% CI)
Unadjusted Models	
Model 1	
Mother ADHD risk	-9.187 (-15.473, 2.901)**
Father ADHD risk	− 0.785 (-6.962, 5.392)
	R^2^ =0.066
	*p*=.015
Adjusted Models	
Model 2	
Mother’s ADHD risk	-9.488 (-15.937, -3.039)**
Father’s ADHD risk	-1.557 (-7.887, 4.772)
Dummy ADHD	1.170 (-4.431, 6.772)
Dummy subthreshold ADHD symptoms	3.441 (-4.399, 11.281)
	R^2^ =0.102
	*p*=.113
Model 3	
Mother’s ADHD risk	-7.694 (-14.507, − 0.881)*
Father’s ADHD risk	-1.455 (-7.732, 4.823)
Dummy ADHD	1.428 (-4.145, 7.000)
Dummy subthreshold ADHD symptoms	4.361 (-3.467, 12.188)
Mother’s psychological difficulties	-5.107 (-10.866, 0.652)
Father’s psychological difficulties	-1.063 (-7.054, 4.928)
	R^2^ =0.132
	*p*=.075

Multiple lineal regression. Models 2–3: adjusted for sex, age, family socioeconomic status, and total IQ. Quality of Life based on KIDSCREEN-27 total score. IQ: Intelligence Quotient; ADHD: Attention-Deficit/Hyperactivity Disorder. Cut-off scales (0 = below cut-off; 1 = at or above cut-off): parental psychological problems (GHQ-12 ≥ 12) and ADHD risk (ASRS ≥ 4). Dummy ADHD: ADHD (1) and subthreshold ADHD symptoms, comparison group (0); Dummy subthreshold ADHD symptoms: subthreshold ADHD symptoms (1) and ADHD, comparison group (0). Significant differences: *for *p* < .05, ** for *p*⩽0.01 and *** for *p*⩽0.001

## Discussion

This study examined the presence of parental ADHD symptoms of children with ADHD (meeting full DSM-5 criteria, subthreshold ADHD symptoms, ADHD + Autism) and without ADHD or autism (comparison group) and explored their association with children’s emotional and conduct problems and their quality of life (QoL), using a longitudinal design study. Maternal ADHD symptoms were associated with children’s emotional problems and quality of life. In contrast, children’s conduct problems were not related to maternal or paternal ADHD symptoms, but rather to the children’s own ADHD.

### Parental ADHD Symptoms by Child Diagnosis

The findings point to a high prevalence of ADHD symptoms and psychological difficulties in parents of children with ADHD. 28.2% of participants had at least one parent reporting ADHD symptoms, consistent with clinical reports showing parental prevalence rates ranging from 33% to 44% among parents with children with ADHD (Agha et al., 2020; Schlarb et al., 2016), and reinforcing the need to systematically assess parental psychopathology during child ADHD evaluations, as untreated parental ADHD may affect the child’s developmental trajectory.

Beyond these findings, parental ADHD prevalence varied across child diagnostic groups. Consistent with previous research (Uchida et al., 2023), parents of children with ADHD self-reported higher rates of ADHD symptoms compared to parents in the comparison group. This pattern was especially pronounced in mothers of children with ADHD + Autism (46.2%) and with clinical ADHD (24.8%), compared to the comparison group (14.7%). These findings align with prior evidence indicating that ADHD traits are more prevalent among parents of children with ADHD than in control families (Agha et al., 2020; Insa Pineda et al., 2020; Vélez-van-Meerbeke et al., 2017), especially in mothers. Our results support the hypothesis that intergenerational transmission of ADHD is more likely when the mother presents symptoms of the disorder (Solberg et al., 2021). In addition, the higher rates of ADHD symptoms observed among mothers of children with comorbid ADHD and autism reinforce the growing evidence of shared heritable and environmental mechanisms across neurodevelopmental conditions, consistent with overlapping genetic risk factors and phenotypic correlations between the two disorders (Canals et al., 2024; Mattheisen et al., 2022). These findings are in line with previous evidence indicating that, at the family level, maternal ADHD symptoms have been associated with elevated autistic traits in offspring (Okyar & Görker, 2020; Van Steijn et al., 2012), supporting the notion of a shared familial vulnerability across neurodevelopmental disorders. However, these findings regarding the ADHD + Autism subgroup should be interpreted with caution due to the small sample size (*n* = 13), which limits statistical power and generalizability of these specific comparisons.

Notably, parents of children with subthreshold ADHD symptoms tended to report slightly higher levels of ADHD symptoms compared to parents in the comparison group, indicating that even subthreshold parental traits may contribute to children’s risk and reinforcing the need to consider ADHD risk on a continuum.

### Parental ADHD Symptoms and Psychological Difficulties in Relation with Children’s Outcomes

Maternal ADHD symptoms and psychological difficulties were independently and significantly associated, both cross-sectionally and longitudinally, with children’s emotional problems and their persistence over time. This is consistent with the broader pattern observed in parents themselves. Both fathers and mothers, who self-report ADHD symptoms, present higher levels of mental health problems, consistent with previous research showing increased stress, anxiety, and depression in both general and clinical populations (Choi et al., 2022). Importantly, parental mental health difficulties have been shown to negatively affect children’s emotional and behavioral well-being (Ribas et al., 2024). In line with these findings, from the cross-sectional perspective, children’s emotional problems were mainly associated with maternal emotional state, underscoring the role of situational and affective factors within the family context in children’s psychological adjustment (Lotto et al., 2024). From the longitudinal perspective, both maternal ADHD symptoms and psychological difficulties were independently and significantly associated with the persistence of children’s emotional problems over time, suggesting that maternal psychological status may contribute to a more stressful caregiving environment, potentially reinforcing difficulties in emotion regulation and less adaptive emotional trajectories in children (Woods et al., 2021). These findings are consistent with previous research linking maternal ADHD symptoms and maternal emotional difficulties with emotional and behavioral problems in offspring (Ingeborgrud et al., 2024; Korja et al., 2024; Levy et al., 2025). One potential mechanism underlying these associations may involve parenting processes. Previous studies have shown that parental ADHD symptoms are associated with greater parenting stress, less consistent parenting practice, and more difficulties in emotion regulation during parent-child interactions (Agha et al., 2013; Johnston & Chronis-Tuscano, 2017). Although parenting variables were not assessed in the present study, these family-level factors, may partly explain why maternal ADHD symptoms were associated with persistent emotional difficulties in children over time.

In addition, the longitudinal design of the present study extends the existing evidence by providing information on the stability of these associations over time, whereas previous studies have been based primarily on cross-sectional designs. Notably, while maternal ADHD symptoms showed consistent longitudinal associations with children’s emotional problems and QoL, neither paternal ADHD symptoms nor paternal psychological difficulties were associated with children’s emotional outcomes. This asymmetry may reflect mothers’ greater involvement in daily caregiving and child management, which could amplify the impact of their psychological functioning on children’s emotional environment. Importantly, these associations are likely bidirectional in nature. Children’s emotional problems may in turn increase maternal stress and psychological burden, creating a self-reinforcing cycle that sustains difficulties over time. This supports developmental models emphasizing reciprocal influences between parental psychopathology and child emotional functioning (Koutra et al., 2025), and underscores the clinical relevance of addressing both maternal and child difficulties simultaneously rather than treating them as independent targets.

Otherwise, maternal ADHD risk was significantly associated with poorer children’s QoL in the regression models, even after controlling for parental psychological difficulties and other psychosocial factors, although no significant differences across diagnostic groups were observed in the univariate analyses. This suggests that the impact of maternal ADHD on child well-being extends beyond genetic transmission, influencing parents’ perceptions of their children’s QoL, emotional state and behavior. This discrepancy with previous studies (Quintero et al., 2017; Wanni et al., 2023) may be partly attributable to methodological differences, as prior studies relied primarily on self-reported measures and clinical samples, whereas the present study used parent-reported QoL in a longitudinal epidemiological cohort.

Unlike the overall associations observed for children’s emotional problems, conduct problems were related to the child’s own ADHD diagnosis, which is consistent with the behavioral and self-regulatory difficulties that characterize ADHD (Faraone et al., 2019; Y. I. Baamer, 2025) and its frequent comorbidity with conduct problems (Gnanavel et al., 2019). Presence of parental ADHD did not have an impact on increases in conduct problems over time. However, the child’s own level of ADHD did have an impact. Children with high levels of ADHD symptoms at EPINED, showed higher levels of conduct problems at EPINED 2. Notably, only the ADHD diagnosis predicted the persistence of conduct problems across both time points, suggesting that core difficulties associated with ADHD, such as impulsivity, may directly contribute to the consolidation of conduct-related problems, regardless of other socio-familial covariates (Ahmad & Hinshaw, 2017). This pattern suggests that conduct problems are more closely linked to neurobiological factors intrinsic to the child’s ADHD, whereas emotional well-being appears to be more sensitive to the family environment, which is influenced by the parents’ psychopathology.

### Limitations and Future Directions

Several limitations should be considered when interpreting the findings. First, the sample size decreased at follow-up, and the small size of certain subgroups, particularly ADHD + Autism group (*n* = 13), reduced statistical power and limits the robustness and interpretability of findings for this subgroup. Second, although the questionnaires were distributed to families as a unit, it was not recorded which parent completed them, which limits the interpretation of sex-specific findings. In addition, the EPINED 2 design may have introduced selection bias, as not all schoolchildren without risk of psychological problems were reassessed at the follow-up phase. Furthermore, the comparison group (without ADHD or autism), was also screened for risk of emotional problems, which may have increased the levels of emotional symptoms in this group and potentially reduced the observed differences between groups.

Future research should aim to increase the size of the sample and to include the children’s self-reports. It would also be valuable to incorporate contextual and family functioning variables, including parenting style and parent-child relationship quality, to better understand the mechanisms that mediate the relationship between parental ADHD symptoms and children’s emotional and conduct difficulties. Our findings underscore the importance of considering maternal psychological well-being in the clinical care of children with neurodevelopmental disorders. Addressing parental mental health may contribute not only to improving parents’ own functioning and family relationships, but also to promoting more favorable developmental outcomes in their children.

### Conclusion and Clinical Implications

This study is the first in Spain to examine ADHD symptoms in mothers and fathers of children with and without ADHD and one of the few to explore their longitudinal associations with children’s mental health and quality of life. Beyond documenting the presence of parental ADHD symptoms, our findings highlight their clinical relevance, as maternal ADHD symptoms are associated with children’s emotional well-being and quality of life, underscoring the importance of assessing and addressing parental ADHD in interventions aimed at improving child outcomes. Our results reveal the high occurrence of ADHD symptoms among mothers of children with ADHD and related conditions, underscoring the intergenerational and dimensional nature of the disorder. Maternal ADHD symptoms and emotional difficulties were strongly associated with children’s emotional problems and lower quality of life over time. Conduct difficulties, in contrast, were primarily related to the child’s clinical ADHD diagnosis.

Identifying ADHD risk and psychological difficulties in parents, particularly mothers, can help tailor more integrated and family-centered intervention approaches, with potential benefits for long-term well-being of both children and parents.

## Supplementary Information

Below is the link to the electronic supplementary material.

Supplementary File 1 (DOCX 28.7 KB)

## Data Availability

Due to legal reasons and the ethical permit for the study, the data that support the findings of this study are not publicly available. The data are accessible on reasonable request from the corresponding authors.
