# Supplementary material for: ADHD Symptoms in Parents of Children With or Without ADHD: Prospective Association With Children’s Quality of Life and Emotional and Conduct Problems (EPINED Cohort)
Source: Res Child Adolesc Psychopathol. 2026 Sep 23;54(5):113. doi: 10.1007/s10802-026-01510-6 (PMC13597650; doi:10.1007/s10802-026-01510-6)
Supplement: Supplementary file 1 [file 10802_2026_1510_MOESM1_ESM.docx]

**SUPPLEMENTARY INFORMATION**

| **Supplementary Table 1.** Occurrence of Psychological Problems in Parents by ADHD Risk | | | |
| --- | --- | --- | --- |
|  | **Parental ADHD Risk** | | |
| **GHQ-12 total scale** | **Risk** | **Without risk** | ***p*** |
| *Mothers* | n=81 | n=364 |  |
| Mean (*SD*) | 12.2(5.9) | 9.4(4.7) | **<.001** |
| Risk (≥12), % (n) | 39.5(32) | 25.5(93) | **.011** |
| *Fathers* | n=58 | n=305 |  |
| Mean (*SD*) | 10.2(3.6) | 8.7(3.7) | **.006** |
| Risk (≥12), % (n) | 29.3(17) | 19.0(58) | .076 |
| ADHD: Attention-Deficit/Hyperactivity Disorder; GHQ-12: General Health Questionnaire Short Form; Parental ADHD Risk = Adult ADHD Self-Report Scale (ASRS) score ≥4 SD: Standard Deviation. Significant differences: *for p<.05, ** for p⩽0.01 and *** for p⩽0.001. | | | |
|  |  |  |  |

| **Supplementary Table 2:** Correlation between variables | | | | | | | | |  | |  | |  | |  |  | |  | |  | |  | |  | |  | |  | |
| --- | --- | --- | --- | --- | --- | --- | --- | --- | --- | --- | --- | --- | --- | --- | --- | --- | --- | --- | --- | --- | --- | --- | --- | --- | --- | --- | --- | --- | --- |
|  | **PEARSON CORRELATION** | | | | | | | | | | | | | | | | | | | | |  | | | | | | | |
|  | **PARENTAL VARIABLES** | | | | | | | | **CHILDREN VARIABLES** | | | | | | | | | | | | | | | | | | | | |
| **Parental variables** | **Mother Age** | **Father Age** | **Parents Separated** | **SES** | **Mother ADHD (ASRS Total)** | **Father ADHD (ASRS Total)** | **Mother PD (GHQ-12 total)** | **Father PD (GHQ-12)** | **Age** | **Sex** | | **Total IQ** | | **Internalizing Problems (CBCL subscale)** | | | **Externalizing Problems (CBCL Subscale)** | | **Emotional Difficulties (SDQ subscale)** | | **Conduct Difficulties (SDQ Subscale)** | | **Emotional problems (Persistence)** | | **Conduct Problems (Persistence)** | | **Quality of Life (KIDSCREEN-27 Score)** | |  |
| **Mother Age** | 1 |  |  |  |  |  |  |  |  |  | |  | |  | | |  | |  | |  | |  | |  | |  | |  |
| **Father Age** | .693*** | 1 |  |  |  |  |  |  |  |  | |  | |  | | |  | |  | |  | |  | |  | |  | |  |
| **Parents Separated** | -.091 | -.071 | 1 |  |  |  |  |  |  |  | |  | |  | | |  | |  | |  | |  | |  | |  | |  |
| **SES** | -.145** | -.038 | .233** | 1 |  |  |  |  |  |  | |  | |  | | |  | |  | |  | |  | |  | |  | |  |
| **Mother ADHD (ASRS Total)** | -.034 | -.054 | .063 | -.010 | 1 |  |  |  |  |  | |  | |  | | |  | |  | |  | |  | |  | |  | |  |
| **Father ADHD (ASRS Total)** | -.034 | -.058 | .043 | -.078 | .489*** | 1 |  |  |  |  | |  | |  | | |  | |  | |  | |  | |  | |  | |  |
| **Mother PD (GHQ-12 total)** | -.024 | -.074 | .103* | .086 | .281*** | .149** | 1 |  |  |  | |  | |  | | |  | |  | |  | |  | |  | |  | |  |
| **Father PD (GHQ-12)** | -.031 | -.032 | -.007 | .083 | .287*** | .232*** | .407*** | 1 |  |  | |  | |  | | |  | |  | |  | |  | |  | |  | |  |
| **Children Variables** |  |  |  |  |  |  |  |  |  |  | |  | |  | | |  | |  | |  | |  | |  | |  | |  |
| **Age** | 3.77*** | .382*** | .008 | -.032 | -.067 | -.024 | .027 | .056 | 1 |  | |  | |  | | |  | |  | |  | |  | |  | |  | |  |
| **Sex** | -.036 | -.007 | -.009 | -.035 | .022 | .028 | .040 | .007 | -.046 | 1 | |  | |  | | |  | |  | |  | |  | |  | |  | |  |
| **Total IQ** | .180*** | .181*** | -.087 | -.245*** | -.019 | .029 | -.039 | -.051 | .051 | .073 | | 1 | |  | | |  | |  | |  | |  | |  | |  | |  |
| **Internalizing Problems (CBCL subscale)** | -.128** | -.142** | .099* | .188*** | .405*** | .373*** | .310*** | .222*** | -.101 | -.008 | | -.126** | | 1 | | |  | |  | |  | |  | |  | |  | |  |
| **Externalizing Problems (CBCL Subscale)** | -.166*** | -.166*** | .126** | .211*** | .318*** | .275*** | .311*** | .228*** | -.227*** | -.048 | | -.249*** | | .591*** | | | 1 | |  | |  | |  | |  | |  | |  |
| **Emotional Difficulties (SDQ subscale)** | -.032 | -.060 | .090 | .144** | .265*** | .149* | .401*** | .145* | -.019 | .157** | | -.116* | | .366*** | | | .228*** | | 1 | |  | |  | |  | |  | |  |
| **Conduct Difficulties (SDQ Subscale)** | -.167** | -.086 | .157** | .162** | .205*** | .154** | .280*** | .148* | -.134* | -.091 | | -.176*** | | .252*** | | | .517*** | | .397*** | | 1 | |  | |  | |  | |  |
| **Emotional problems (Persistence)** | .002 | -.053 | .061 | .064 | .186*** | .168** | .247*** | .055 | -.067 | .096 | | -.029 | | .410*** | | | .204*** | | .552*** | | .172*** | | 1 | |  | |  | |  |
| **Conduct Problems (Persistence)** | -.197*** | -.192*** | .089 | .103 | .188*** | .192*** | .219*** | .097 | -.167** | -.078 | | -.110* | | .323*** | | | .535*** | | .162** | | .582*** | | .143** | | 1 | |  | |  |
| **Quality of Life (KIDSCREEN-27 Score)** | -.016 | -.034 | .033 | -.023 | -.309*** | -.116 | -.321*** | -.167 | -.155* | -.068 | | .039 | | -.278*** | | | -.109 | | -.330*** | | -.236*** | | -.251*** | | -.074 | | 1 | |  |
| **Abbreviations:** SES: Socioeconomic Status; PD: Psychological Difficulties; ADHD: Attention-Deficit/Hyperactivity Disorder; ASRS: Adult ADHD Self-Report Scale; GHQ-12: General Health Questionnaire Short Form; IQ: Intelligence Quotient; CBCL: Child Behavior Checklist; SDQ: Strengths and Difficulties Questionnaire. Significant differences: *for p<0.05, ** for p⩽0.01 and *** for p⩽0.001. | | | | | | | | | | | | | | | | | | | | | | | | | | | | | |
